# Clinical Impact of Multi-omics profiling of extracellular vesicles in cancer Liquid Biopsy

**DOI:** 10.1016/j.jlb.2024.100138

**Published:** 2024-01-04

**Authors:** Mrunal Kulkarni, Rishav Kar, Srestha Ghosh, Swarup Sonar, Divya Mirgh, Indra Sivakumar, Abhijit Nayak, Raman Muthusamy

**Affiliations:** a Department of Pharmacy, BITS Pilani, Rajasthan, 333031, India; b School of Biological Sciences, Ramakrishna Mission Vivekananda Educational and Research Institute (RKMVERI), Kolkata, 700026, India; c Department of Microbiology, Lady Brabourne College, Kolkata, West Bengal, 700017, India; d Genpact, Badshahpur, Sector 69, Gurugram, Haryana, 12210, India; e Department of Infectious Diseases, Vaccine and Immunotherapy Center, Massachusetts General Hospital, Boston, MA, USA; f Dept of Medical Microbiology, Saveetha Medical College and Hospitals, SIMATS, Chennai, 602105, Tamil Nadu, India; g Institute of Dental Sciences, SOA University, Bhubaneswar, Odisha, 751003, India; h Department of Oral Medicine and Radiology, Saveetha Dental College and Hospitals, Saveetha Institute of Medical and Technical Sciences, Chennai, 600077, India; i Center for Global Health Research, Saveetha Medical College & Hospitals, Saveetha Institute of Medical and Technical Sciences, Chennai, 602105, Tamil Nadu, India

**Keywords:** Exosomes, Cancer, Biomarkers, Multi-omics

## Abstract

Extracellular vesicles (EVs) are the cell's secreted component. It is majorly classified into microvesicles, apoptotic bodies, and exosomes. Exosomes play a significant role in cancer development and progression. Its molecular signature (DNA, RNA, Proteins, lipids) has more priority in cancer profiling current decade. In cancer prevention, the most challenging part is early detection. EVs-based cancer screening develops a promising platform. Multi-Omics exosomes profiling-based liquid biopsy support early cancer detection more efficient way. This approach provides detailed molecular expression data (it may be inner cargos or surface express molecules). This article highlited multi-omic exosome profiling-based exosome theranostics applications in cancer, technical challenges, and improvisation for future improvement.

## 1 Introduction

**Fig. 1:**
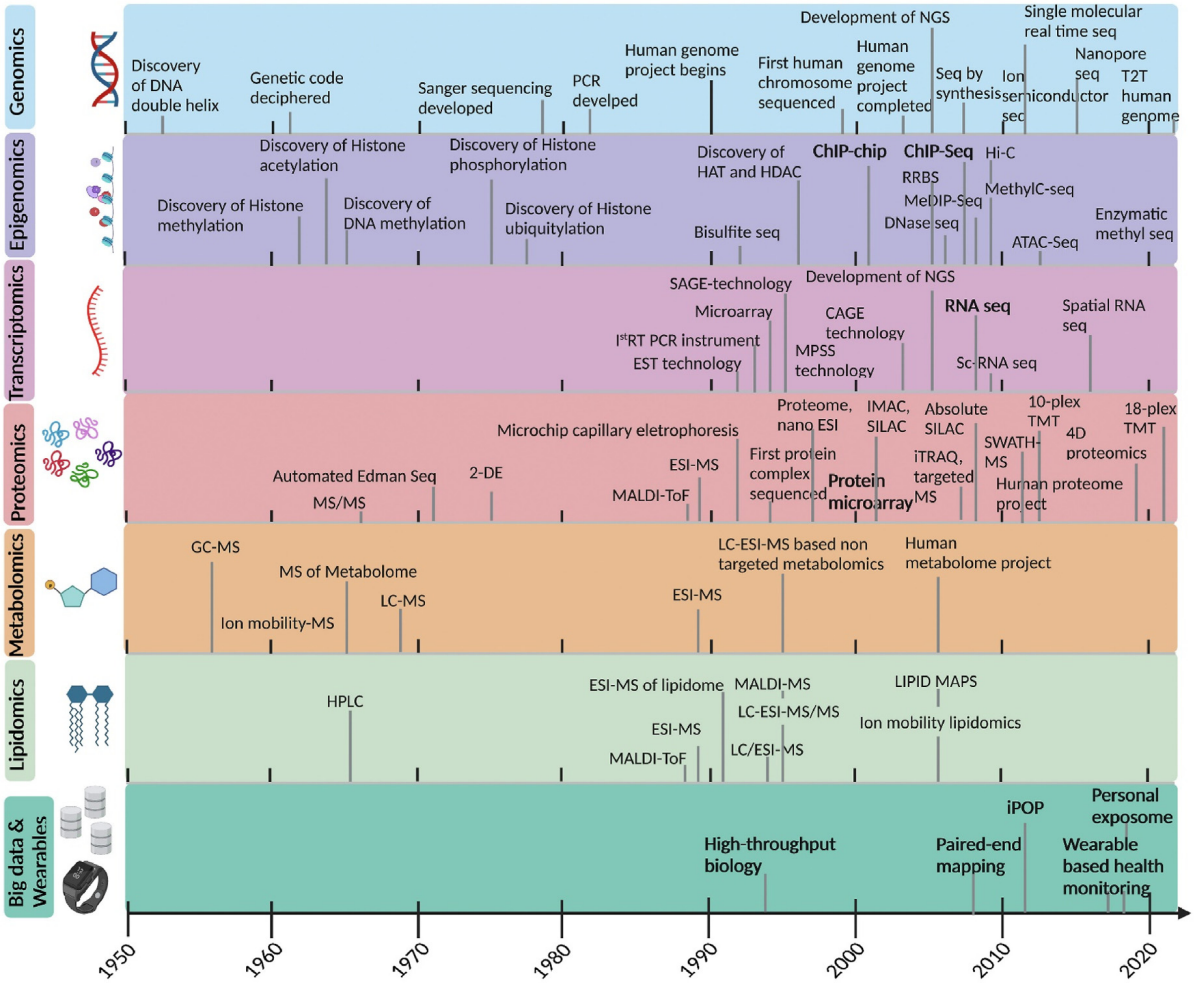
The landscape of omics analysis (2-DE, 2-Dimensional Electrophoresis; ATAC, Assay for Transposase-Accessible Chromatin; CAGE, Cap Analysis Gene Expression; DNMT1, DNA Methyl Transferase one; ESI, Electrospray Ionization; EST, Expressed Sequence Tags; GC, Gas Chromatography; HAT, Histone Acetyl Transferase; HDAC, Histone Deacetylase; Hi-C, High-throughput Chromosome Conformation Capture; HPLC, High Performance Liquid Chromatography; IMAC, Immobilized Metal Affinity Chromatography; iTRAQ, Isobaric Tags for Relative and Absolute Quantitation; LC, Liquid Chromatography; MALDI, Matrix-Assisted Laser Desorption/Ionization; MeDIP, Methylated DNA Immunoprecipitation; MPSS, Massively Parallel Signature Sequencing; MS, Mass spectrometry; NGS, Next Generation Sequencing; PCR, Polymerase Chain Reaction; SAGE, Serial Analysis of Gene Expression; Sc-RNA, Single cell RNA; SILAC, Stable Isotope Labeling with Amino acids in Cell culture; T2T, Telomere-To-Telomere**)** (Reproduced with permission under Creative Commons CC BY 4.0 license from ref. [44] Copyright @ 2023 The Authors).

Cancer has emerged as an unformidable, debilitating ailment in contemporary times and claiming numerous lives each year due to the lack of early detection. Approximately 10.0 million cancer-related deaths, excluding non-melanoma skin cancer, and 19.3 million new cancer cases were reported worldwide in 2020. When it comes to new diagnoses, female breast cancer has emerged as the most common type, with around 2.3 million cases (11.7 %), more than colorectal cancer (10.0 %), stomach cancer (5.6 %), lung cancer (11.4 %), and prostate cancer (7.3 %) combined. With an anticipated 1.8 million deaths (18 %), lung cancer continued to top all cancer-related causes of death. Colorectal cancer (9.4 %), liver cancer (8.3 %), stomach cancer (7.7 %), and female breast cancer (6.9 %) were the next most common cancer-related deaths. According to projections, there will be 28.4 million instances of cancer worldwide in 2040, a 47 % increase from 2020. Because of changes in the population, it is anticipated that this surge will be greater in transitioning nations (64 %–95 %) than in transitioned countries (32 %–56 %). Furthermore, the rise could be exacerbated by growing risk factors linked to economic expansion and globalization [1]. Although, it is still difficult to diagnose any type of cancer in its early stages, requiring invasive procedures such as tissue biopsies and colonoscopies. Non-invasive, accurate, and dependable diagnostic instruments are sorely needed worldwide to improve prognosis and early detection [2]. Extracellular vesicles (EVs) offer a promising solution for early detection. Extracellular vesicles (EVs) are membranous structures with a phospholipid bilayer that are released by various cell types. They are present in a variety of biological fluids, including saliva [3], breast milk [4], tears [5], sweat [6], plasma [7], blood, urine [8], cerebrospinal fluid, and others. This process is referred to as “liquid biopsy" and is primarily focused on the early diagnosis of diseases [9]. EVs function as carrier molecules in the body through intercellular communication. Extracellular vesicles (EVs) can be further subdivided into three groups: exosomes that originate from endosomes, apoptotic bodies that originate from the endoplasmic reticulum and the plasma membrane, and microvesicles that derive from the plasma membrane [10]. Exosomes are subpopulations of EVs that play an important role as signaling molecules in detecting cancer at an early stage. Exosomes are a rich source of cargo molecules, for instance, DNA [11], microRNA, long noncoding RNA, and Protein), which regulate cancer metastasis [12]. Tumor-derived EVs, or “TEXs," are involved in cancer metastasis because they can cause hypoxia, unchecked cell growth, angiogenesis, immunosuppression, extracellular matrix (ECM) remodeling, drug resistance, therapy resistance, and the cancer stem cells development [[13], [14], [15]]. In cancer diagnosis, EVs profiling has become the most interesting event for early diagnosis of cancer. Muti-omics-based EVs profiling is a promising cancer biomarker invention phenomenon. Omics analysis regarding technical development, and important inventions are summarized in Fig. 1.

## 2 Multi-omics profiling of EVs in cancer

EVs are the source of cancer biomarkers. Isolation of EVs is done via several methods such as ultracentrifugation, size-exclusion chromatography, gradient centrifugation, co-precipitation method, microfluidic device, etc. [10]. Isolated Exosome are analyzed via Transmission electron microscopy (TEM)- morphology, Scanning electron microscopy (SEM)-surface analysis, Nanoparticle tracking assay (NTA)-size distribution analysis, western blotting-(marker expression, like 10.13039/100011639CD63), electrochemical and aptamer sensor (significant molecular expression based exosome separation), digital droplet PCR (exosome-based cancer mutation identification) [11], Nanopore (based on charge EVs separation, tumor EVs are more negatively charged), Surface plasmon resonance sensor (this method is more sensitive, efficient, and effective compared to enzyme-linked immunosorbent assay (ELISA) for exosome-based cancer biomarkers detection), Raman spectra (for molecular profiling), Fourier-transform infrared spectroscopy (FTIR) (presence of chemical group identification), Flowcytometry (molecular expression identification), Omics profiling of exosome (it support molecular expression based functional activity analysis in cellular system and cancer biomarkers investigation), and artificially intelligence (AI) and machine learning (ML)-use clinical databased analysis [10,41], All this process provide a detailed profiling data about EVs molecule (DNA, RNA, proteins, lipids etc.) which play significant role in cancer detection [16]. EVs cargo molecular diversity depending on cell physiology. Normal cell (mean healthy) EVs are completely different from tumor-derived EVs. Exosome proteome analysis is frequently used to explore the differences in exosomal cargo between normal and malignant cell-generated EVs [17]. In one of the experiments, urine was collected from 10 healthy participants and 8 patients with Non-small cell lung cancer (NSCLC). The proteome differences of the isolated EVs were first evaluated using 1D SDS-PAGE (Sodium Dodecyl Sulphate-Polyacrylamide Gel Electrophoresis). Mass spectrometry was used to sequence the differentially expressed proteins. According to the scientists, 18 proteins were detected in urine exosome preparations, 11 of them were exclusive to NSCLC EVs, 4 to normal EVs, and 3 to both. Leucine-rich alpha-2-glycoprotein 1(LRG1), a well-known serum cancer biomarker [18], was shown to be substantially elevated in NSCLC EVs. The authors propose that LRG1 could be used to diagnose NSCLC using a simple urine test. EVs are a transporter of several biomolecules such as DNA, RNA, proteins, lipids, etc. This molecular cargo has clinical significance (cell messenger of healthy or undergoing pathological complication) and cell-to-cell communication [[19], [20], [21], [22]]. In exosome biology, cargo sorting and packaging mechanisms need to be explored in more detail way. The molecular cargos of exosome is a promising biomarkers source of cancer [23]. The exosome is a key regulator of tumor microenvironment (TME) in promoting cancer [24,25]. The vesicular proteomes gives a wealth of knowledge about the biogenesis mechanisms and pathophysiological activities of extracellular vesicles, as well as aiding in the discovery of biomarkers based on the protein signature of the generating cells [26]. In proteomics, mass spectrometry-based exosome profiling supports several clinically significant proteins expression analyses [27]. In a liquid biopsy, the fusion of EVs and advanced technology (nanotechnology-based isolation, sensor) develop a new cancer diagnostic era [28]. In cancer, exosome surface omics profiling supports specific cancer biomarker detection [29,30]. Lipidomic [31,32] and metabolomic [33,34] profiling of EVs reveals exosome participation in cancer development and cellular communication mechanisms. Both approaches are also significant for cancer screening [35,36].

## 3 Challenges and future prospective multi-omics approach of exosome profiling

Single-point interaction of cause and its effect is presently getting dropped as multi-omic studies are coming into the limelight. But in this domain of research EVs provide a new level of difficulties [37]. Extracellular vesicles are heterogeneous by origin, molecular diversity, and function, and have been placed in an essential place of investigation. It is no longer being only subjected to cell communication, and hence single exosome profiling is continuously getting the attention of today's researchers. But still, the multi-omic approach of exosome profiling has some limitations and improvements which are summarized below in Table 1.

**Table 1:** Multi-omics-based EVs profiling.

Omics approach	Clinical significances	Challenges	Improvement	References
**Genomics**	Cancer Diagnostic and prognostic biomarkers detection Genome Profiling of EVs	EVs Heterogeneity Purity of EVs DNA diversity Tissue-based EVs diversity	Single exosome profiling Exosome barcoding Digital droplet PCR	[[38], [39], [40], [41]]
**Transcriptomics**	Cancer biomarkers Exosome RNA profiling	EVs Heterogeneity RNA diversity Exosome RNA-based gene regulation is not clear	Single exosome profiling Exosome barcoding	[[38], [39], [40]]
**Proteomics**	Cancer biomarkers detection Optimization of Exosome-based drug delivery	EVs Heterogeneity Isolation protocol	Single exosome profiling Exosome barcoding	[[38], [39], [40], [41], [42]]
**Surface omics**	EVs-based therapeutic development	It has limited assessment of the specificity of surface markers of EVs	Single exosome profiling Exosome barcoding Surface modification	[29,[38], [39], [40]]
**Lipidomic**	Cancer biomarkers detection EVs origin	Lack of knowledge of exosome lipids	Single exosome profiling Exosome barcoding	[[38], [39], [40]]
**Metabolomics**	Cancer biomarkers detection Cell metabolic activity understand	Along with science literature, databases of EVs also does not contain enough information about exosome metabolome	Single exosome profiling Exosome barcoding	[[38], [39], [40],^43^]
**Multi-omics**	Cancer biomarkers detection Therapeutic development	Lack of standard protocols EVs Heterogeneity	Single exosome profiling Exosome barcoding Effective omics data analysis tool deployment	[38,41]

With many difficulties and limitations, the multi-omics approach has a very bright future. These approaches will undoubtedly provide much deeper insights into these nano-sized biological entities. If researchers could establish appropriate bioinformatic tools and computational approaches then it will take a multi-omics approach one step closer to its success in terms of data assembly and interpretation. One of the many outcomes of this technology will be the development of vaccines against various cancers. nanomedicines against various infectious diseases and nosocomial-acquired infections. This field is still in its infancy and requires proper nourishment to bear fruits for the benefit of mankind.

## 4 Conclusion

Multi-omics-based cancer profiling is a promising platform for effective cancer theranostics developments. EVs biology requires some time for proper protocol development (isolation with purity, large-scale production, clinical trials for medical application), a deep understanding of exosome biology, and efficient bioinformatic tool development for exosome omics data analysis. Hope in the future it leads to a precision oncology era.

## Ethics approval and consent to participate

Not applicable.

## Consent for publication

Not applicable.

## Data avaibility statement

Not applicable.

## Declaration of competing interest

The authors are declaring no conflict of interests.
